# The *in Vitro* Structure-Related Anti-Cancer Activity of Ginsenosides and Their Derivatives

**DOI:** 10.3390/molecules161210619

**Published:** 2011-12-19

**Authors:** Hang Dong, Li-Ping Bai, Vincent Kam Wai Wong, Hua Zhou, Jing-Rong Wang, Yan Liu, Zhi-Hong Jiang, Liang Liu

**Affiliations:** 1 Shum Yiu Foon Shum Bik Chuen Memorial Centre for Cancer and Inflammation Research, School of Chinese Medicine, Hong Kong Baptist University, 7 Baptist University Road, Kowloon Tong, Hong Kong, China; 2 State Key Laboratory of Quality Research in Chinese Medicine, Macau University of Science and Technology, Avenida Wai Long, Taipa, Macau, China

**Keywords:** ginsenosides, anti-cancer, cytotoxicity, protopanaxadiol, structure-related activity

## Abstract

*Panax ginseng* has long been used in Asia as a herbal medicine for the prevention and treatment of various diseases, including cancer. The current study evaluated the cytotoxic potency against a variety of cancer cells by using ginseng ethanol extracts (RSE), protopanaxadiol (PPD)-type, protopanaxatriol (PPT)-type ginsenosides fractions, and their hydrolysates, which were prepared by stepwise hydrolysis of the sugar moieties of the ginsenosides. The results showed that the cytotoxic potency of the hydrolysates of RSE and total PPD-type or PPT-type ginsenoside fractions was much stronger than the original RSE and ginsenosides; especially the hydrolysate of PPD-type ginsenoside fractions. Subsequently, two derivatives of protopanaxadiol (**1**), compounds **2** and **3**, were synthesized *via* hydrogenation and dehydration reactions of compound **1**. Using those two derivatives and the original ginsenosides, a comparative study on various cancer cell lines was conducted; the results demonstrated that the cytotoxic potency was generally in the descending order of compound **3** > 20(*S*)-dihydroprotopanaxadiol (**2**) > PPD (**1**) > 20(*S*)-Rh2 > 20(*R*)-Rh2 ≈ 20(*R*)-Rg3 ≈ 20(*S*)-Rg3. The results clearly indicate the structure-related activities in which the compound with less polar chemical structures possesses higher cytotoxic activity towards cancer cells.

## 1. Introduction

Cancer, a generic term for a large group of diseases that can affect any part of the body, is a leading cause of death worldwide and accounted for 7.6 million deaths (around 13% of all deaths) in 2008 [1]. The major interventions of conventional medicine include surgery and radio/chemotherapy which aim at eliminating the cancer or prolonging life span of the patients [2]. However, it is often of those interventions failed to reach their expected results. Thus, patients, doctors, and researchers are looking elsewhere for effective therapeutics, particularly from natural sources [3]. 

*Panax ginseng* has become, arguably, the most popular herb for treatment of various diseases, including cancers, in Asia if not across the World. Ginseng extracts and its chemical components have been pronounced capable of reducing risk of cancers, including inhibition of carcinogenesis in oral cavity, stomach, lung, liver, pancreas, ovaries, and colon [4]. The pharmacological properties of ginseng are generally attributed to its component triterpene glycosides, called ginsenosides [5]. Nevertheless, whether the intact ginseng is suitable for *in vitro* anticancer studies and which hydrolysis products could exert anticancer effect remains unclear. Previous reports showed that the degradation and metabolism of glycosides derived from medical plants often affect their pharmacological potency as well as their clinical therapeutic efficacy; these degradation and metabolism processes occur even in the gastrointestinal tract [6]. For instance, the ginsenosides Rb1, Rb2, Rc, Re can be hydrolyzed in artificial gastric fluid (at 37 °C and pH 1.2) [7,8]; this could alter the composition of ginsenosides as well as their corresponding bioactivities both qualitatively and quantitatively [9,10]. It is estimated that the hydrolytes and/or metabolites of ginsenosides may play an important role in anti-cancer *in vivo* when ginsenosides are taken orally. Therefore, we screened the bioactive anti-cancer compounds from ginseng by using hydrolytes of ginseng extracts and ginsenosides rather than using their original extracts and ginsenosides. We expected that using hydrolyzed products of ginseng would more closely mimic the biochemistry of ginseng in patients, and thus results would be more relevant to clinical usage. 

Depending on their aglycone content, ginsenosides are divided into protopanaxadiol (PPD)-type, protopanaxatriol (PPT)-type and oleanane-type ginsenosides. As ginsenosides are subject to metabolism mainly *via* hydrolysis and dehydration processes in the gastrointestinal tract, we therefore evaluated the cytotoxicity of ginseng extract and ginsenosides fractions after a stepwise hydrolysis of the sugar moieties against a variety of cancer cells. To further elucidate the activity-structure relationship of ginsenosides, we synthesized two derivatives (compounds **2** and **3**) of PPD (**1**) and evaluated their cytotoxic effects on a series of cancer cell lines. As a result, these two derivatives **2**, **3** derived from PPD (**1**) were, for the first time, found to have potent anticancer potency, and of these compound 3 demonstrated the highest anticancer activity. 

## 2. Results and Discussion

### 2.1. Hydrolysate of Ginseng Extracts Preparation and Its Cytotoxicity

**Figure 1:**
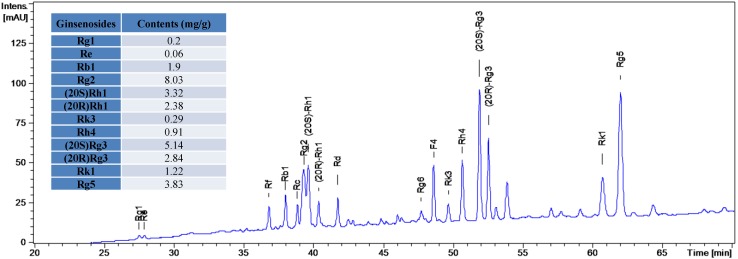
LC-UV chromatograms of the RSE hydrolysates (RSEH).

The ethanol extract of ginseng (RSE) was hydrolyzed in artificial gastric juice to prepare the hydrolysates of RSE (RSEH) for the study. Briefly, the dried RSE (20 g) was dissolved in the artificial gastric juice (0.1 N HCl, 1,800 mL) and then subjected to incubation in a VK7025 Dissolution Tester for 2 h (Temp: 37 ± 0.5 °C; Sprindle speed: 100 rpm). The hydrolysis solution was subsequently applied to freeze-dry after being neutralized with 10 M NaOH to yield RSEH (32.7 g, the content of NaCl is 38.8%). The composition and contents of RSEH were analyzed by HPLC-TOF-MS. By comparing the retention times with authentic compounds, primary and secondary ginsenosides (de-glycosylated ginsenosides) could be identified (Figure 1). The results showed that the secondary ginsenosides such as Rg3, Rk1 and Rg5 accounted for the majority of the constituents in RSEH.

**Figure 2:**
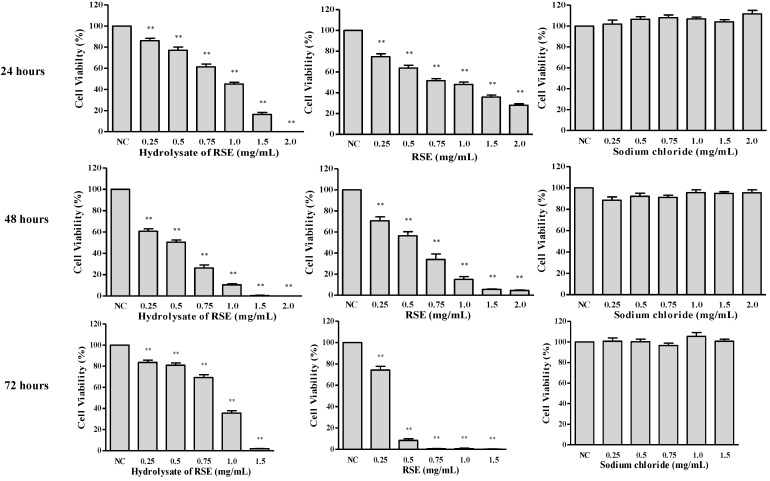
Time- and dose-dependent cytotoxicity of RSEH and RSE on LLC1 cells.

The cytotoxic potency of RSEH and RSE against LLC1 cells with different drug concentrations and treatment time points was compared. Significant and dose-dependent cytotoxicity was observed when LLC1 cells were exposed to RSEH or RSE for 24 to 72 h. The results showed that RSEH exhibited higher cytotoxic effects than RSE after 24 and 48 h, but when LLC1 cells were treated with RSEH or RSE for 72 h, their cytotoxic effects were reversed, *i.e.*, RSE demonstrated much stronger cytotoxic effect than RSEH (Figure 2). This could be explained by the fact that RSEH may be not as stable as the RSE form in the cell culture medium condition, and as a result, the cancer cells might be able to metabolize or detoxify the RSEH after 48 h treatment (t = 48 h). In the case of the longer treatment period, e.g., (t = 72 h), the remaining cancer cells may start to proliferate again and show increased cell viability after 24 h during the 72 h MTT assay, and as a result, the cytotoxic effect of RSEH at 72 h seem to be weaker compared to its cytotoxic effect at 48 h.

### 2.2. Hydrolysate of PPD- or PPT-Type Ginsenosides Fraction Preparation and Its Cytotoxicity

**Figure 3:**
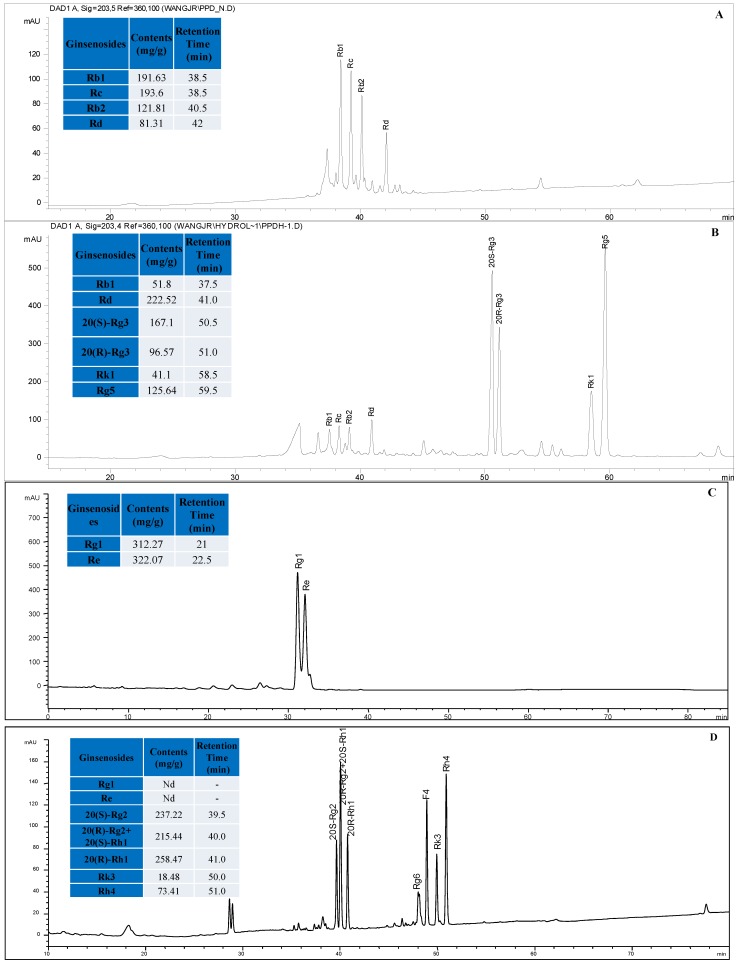
HPLC chromatograms of the PPD-type ginsenosides fraction (**A**) and its hydrolysate PPDH (**B**), PPT-type ginsenosides fraction (**C**) and its hydrolysate PPTH (**D**).

The PPD- or PPT-type ginsenosides fraction prepared from RSE (250 mg) were dissolved in 300 and 100 mL artificial gastric acid (0.1 N HCl) and incubated in a Julabo Shake Temp SW 22 circulating water bath at 37.5 °C. The hydrolysate solutions were neutralized with NaOH and subsequently subjected to liquid-liquid extraction with *n*-BuOH (45 mL × 3). The *n*-BuOH extracts were evaporated under reduced pressure, and the residues were freeze-dried to yield hydrolysates of the PPD-type ginsenosides fraction (PPDH, 167 mg) and the PPT-type ginsenosides fraction (**PPTH**, 150 mg). Typical LC-UV chromatograms of **PPDH** and PPTH are shown in Figure 3. Contents of twelve ginsenosides in the PPDH and PPTH were further determined by using an HPLC-UV method.

**Figure 4:**
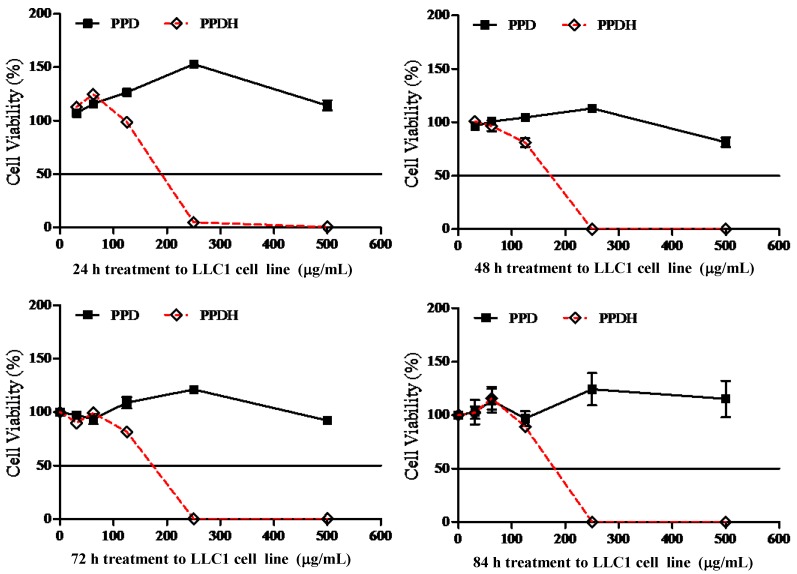
Time and dose-dependent cytotoxicity of PPD and PPDH on LLC1 cells.

We next examined the cytotoxic potency of the PPDH and PPTH ginsenosides against several cancer cell lines. At the concentrations of 250 μg/mL and 500 μg/mL, PPDH ginsenosides were able to reduce the cell survival rates of LLC1 cells at different treatment time periods, but no time-dependent relationship was found (Figure 4). 

**Figure 5:**
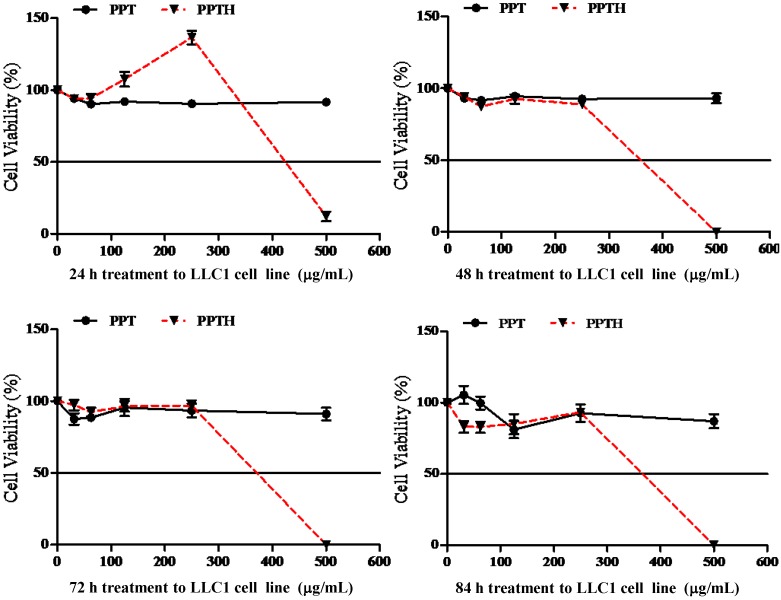
Time- and dose-dependent cytotoxicity of PPT and PPTH on LLC1 cells.

The IC_50_ value of the PPD-type ginsenosides fraction decreased from 750 μg/mL (data not shown) down to 180 μg/mL in LLC1 cells after acid hydrolysis. Although the PPT-type ginsenosides fraction displayed no cytotoxic effects, PPTH ginsenosides could reduce cell survival rate of LLC1 cells at a drug concentration of 500 μg/mL, but without any time-dependent relationship (Figure 5).

**Figure 6:**
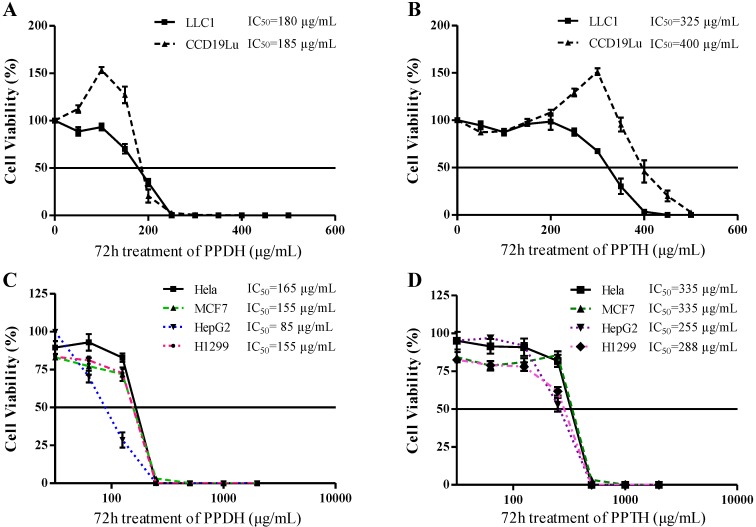
Effect of PPDH or PPTH ginsenosides on inhibition of cell growth of the lung normal fibroblast cell line CCD19Lu and cancerous cell lines.

In order to prove whether this cytotoxic effect was specific to tumor cells, we further compared the effect of PPDH and PPTH ginsenosides on cell viability using the LLC1 and lung normal fibroblast cell line, CCD19Lu. The results showed no obvious differences of the cytotoxic potency in PPDH-treated LLC1 and CCD19Lu cells; the IC_50_ on those two cell lines were 180 μg/mL and 185 μg/mL, respectively (Figure 6A). Likewise, IC_50_ values of PPTH ginsenosides on LLC1 and CCD19Lu cells showed no marked differences, *i.e.*, 325 μg/mL and 400 μg/mL, respectively (Figure 6B). In addition, as shown in Figure 6C and Figure 6D, PPDH and PPTH ginsenosides displayed similar cytotoxic potencies against four types of cancer cell lines with different genetic backgrounds. Taken together, these results indicate that although the cytotoxic effects of total PPD- and PPT-type ginsenosides fractions were increased after hydrolysis, both almost equally impaired normal lung fibroblasts.

### 2.3. Examination of the Cytotoxic Potency of PPD-type Ginsenosides and their Structurally-Modified PPD-Derivatives

**Figure 7:**
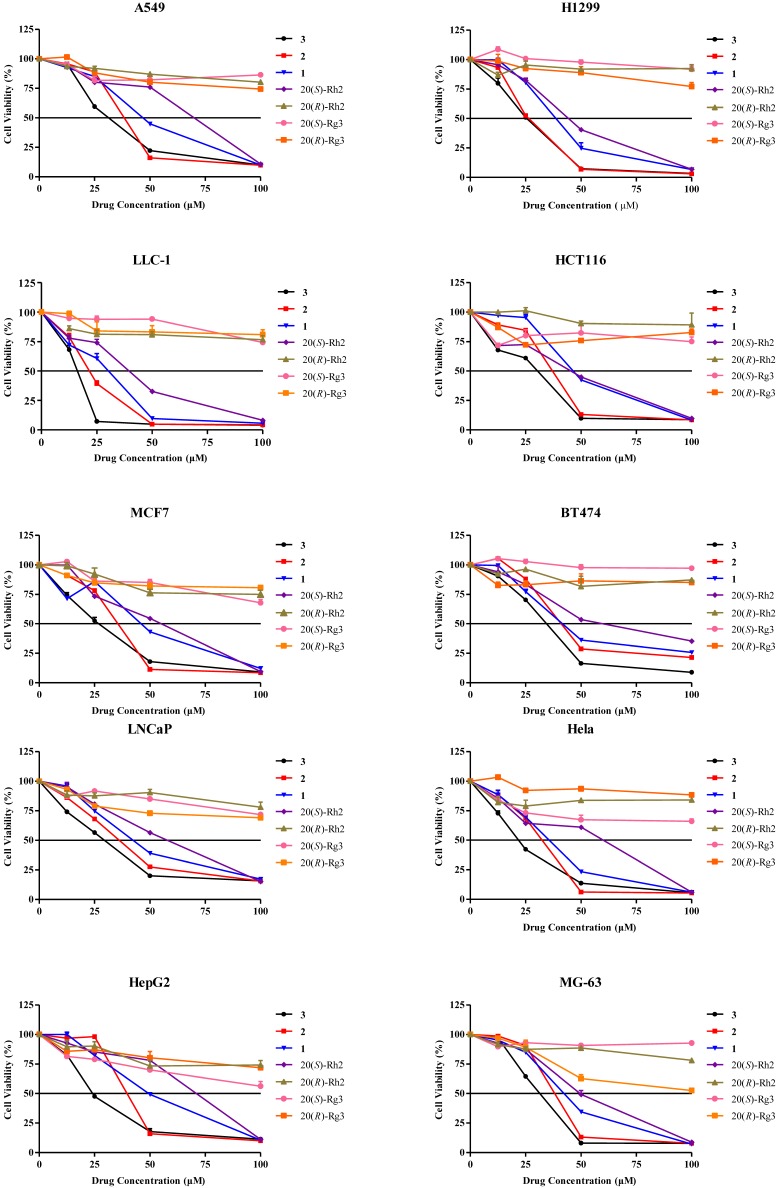
Cytotoxicity of PPD-type ginsenosides and PPD-derivatives against various cancer cell lines.

**Figure 8:**
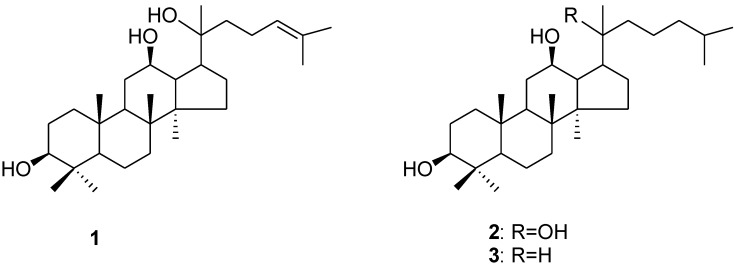
Chemical structures of PPD (**1**) and its synthetic derivatives **2** and **3**.

Numerous investigations have revealed that the anticancer activity of ginseng can be predominantly attributed to PPD-type ginsenosides, in comparison to the PPT-type ginsenosides. Accordingly, we examined the cytotoxic activity of PPD-type secondary ginsenosides, including Rg3, Rh2 and the aglycone 20(*S*)-PPD, in ten cancer cell lines representing seven types of clinical malignancies. 20(*S*)-Rh2 and 20(*R*)-Rg3, which are currently marketed as anticancer agent and dietary supplement, respectively, for cancer patients in China, were used as controls. As shown in Figure 7, the cytotoxic potencies of PPD-type ginsenosides showed a descending trend of PPD > Rh2 > Rg3, indicating that increase in bioactivity correlated with decrease of the molecular polarity. According to this finding, we speculated that cytotoxic potency might be further enhanced along with increase of the lipophilicity of PPD (**1**). In order to verify this hypothesis and obtain more potent cytotoxic compounds, two PPD-derivatives, 20(*S*)-dihydroprotopanaxadiol (**2**) [11,12] and **3** (Figure 8) [13], were synthesized using PPD (**1**) as raw material. The PPD-derivative **2** was obtained by reducing the double bond in the side chain; while the PPD-derivative **3** was obtained by hydrogenation of double bonds after a dehydration reaction at the C-20 position of PPD (**1**). The cytotoxic activities of these two structurally-modified PPD derivatives were subsequently examined on the same cancer cell lines and compared with PPD (**1**), Rg3 and Rh2. As demonstrated in Figure 7, 20(*S*)-Rh2, PPD, 20(*S*)-dihydroprotopanaxadiol and the PPD-derivative **3**, with gradual deglycosylation, hydrogenation and dehydration, exhibited different potencies on inhibition of the cancer cell lines growth in a dose-dependent manner. Using the same drug concentrations of the tested compounds in the experiments, 20(*R*)-Rh2, 20(*S*)-Rg3 and 20(*R*)-Rg3 showed low cytotoxic potency to the tested cancerous cell lines, although Rg3 was reported effective for anti-cancer in the *in vivo* experiments [14,15,16]. The cytotoxic activity generally occurred in the order of: compound **3** > 20(*S*)-dihydroprotopanaxadiol (**2**) > PPD (**1**) > 20(*S*)-Rh2 > 20(*R*)-Rh2 ≈ 20(*R*)-Rg3 ≈ 20(*S*)-Rg3. For PPD-type ginsenosides, increasing cytotoxic effect was correlated with decrease of the sugar numbers in their chemical structures. As a whole, for PPD derivatives, more lipophilic chemical structures appear to confer more potent cytotoxicity with regard to cancer cells.

## 3. Experimental

### 3.1. Instruments and Materials

^1^H-NMR spectra were recorded using a Bruker-400 NMR spectrometer, and chemical shifts are presented in δ (ppm) relative to Me_4_Si used as internal standard. High resolution mass spectroscopy measurements were performed on a Q-TOF mass spectrometer (Bruker Daltonics, Billerica, MA, USA). 20(*S*)-Protopanaxadiol was purchased from Shanghai PharmValley Corporation (Shanghai, China).

### 3.2. HPLC Analysis of Ginsenosides in RSEH, PPTH and PPDH

HPLC analyses of ginsenosides in RSEH, PPTH and PPDH were performed on an Agilent (Palo Alto, CA, USA) 1100 series HPLC consisting of a binary pump, an automatic sample injector, and a diode array detector. The samples were separated on a Zorbax Eclipse XDB-C18 column (Agilent, 4.6 mm × 250 mm, 5 μm) at 25 °C with a sample injection volume of 20 μL. The mobile phase was a gradient of water (A) and acetonitrile (B), starting isocratically with 20% B for 20 min and increasing to 46% B at 20–45 min, then increasing to 55% B at 45–60 min, and finally to 100% at 60–105 min. The flow rate of the mobile phase was 1.0 mL/min, and the detector wavelength was 203 nm. 

### 3.3. Synthesis of the PPD (**1**) Derivatives **2** and **3**

*20(S)-Dihydroprotopanaxadiol* (**2**): 20(*S*)-Protopanaxadiol (**1**, 210 mg) in methanol (20 mL) was stirred under a H_2_ atmosphere at room temperature for 3 hr in the presence of palladium hydroxide on carbon powder (58 mg). The product was purified by ODS column chromatography eluted with a gradient from 80% aqueous to 100% methanol to yield PPD-derivative **2** (180 mg). High Resolution ESI-MS (Positive ion mode): *m/z* 485.3978 [M+Na]^+^ (calculated for C_30_H_54_NaO_3_: 485.3965). ^1^H-NMR (CDCl_3_): 3.60 (1H, td, *J* = 10.4, 5.2 Hz, H-12), 3.20 (1H, dd, *J* = 11.0, 4.0 Hz, H-3), 2.04 (1H, td, *J* = 10.8, 7.0 Hz, H-17), 1.18 (3H, s, H-21), 0.99 (3H, s, H-18), 0.98 (3H, s, H-28), 0.89 (3H, s, H-30), 0.88 (6H, d, *J* = 6.3 Hz, H-26 and H-27), 0.88 (3H, s, H-19), 0.78 (3H, s, H-29), 0.73 (1H, d, *J* = 10.8 Hz, H-5).

Derivative (**3**) was obtained by the following procedure: 20(*S*)-protopanaxadiol (**1**, 500 mg) in DMSO (8 mL) was heated at 140 °C in an oil bath for 50 min. The reaction solution was directly loaded to an ODS column to give the dehydrated products of PPD (350 mg). The dehydrated products (330 mg) in methanol (25 mL) were subjected to catalytic hydrogenation reaction under a H_2_ atmosphere in the presence of palladium hydroxide on carbon powder (66 mg). The reaction solution was dried and purified over an ODS chromatography column using an gradient from 90% aqueous to 100% methanol as eluent to yield compound **3**. High Resolution ESI-MS (Positive ion mode): *m/z* 429.4067 [M-H_2_O+H]^+^ (calculated for C_30_H_53_O: 429.4091), 447.4162 [M+H]^+^ (calculated for C_30_H_55_O_2_: 447.4197). ^1^H-NMR (CDCl_3_): 3.63 (1H, td, *J* = 10.4, 5.2 Hz, H-12), 3.20 (1H, dd, *J* = 11.32, 5.0 Hz, H-3), 0.99 (3H, s, H-18), 0.96 (3H, s, H-28), 0.90，0.80 (3H, d, *J* = 6.8 Hz, H-21), 0.88 (3H, s, H-30), 0.87 (6H, d, *J* = 6.8 Hz, H-26 and H-27), 0.87 (3H, s, H-19), 0.78 (3H, s, H-29), 0.72 (1H, dd, *J* = 11.1, 1.8 Hz, H-5).

### 3.4. Cancer Cell Lines and Cell Culture Conditions

All cancer cells were acquired from the American Type Culture Collection (Rockville, MD, USA) and cultured according to their guidelines.

### 3.5. Cytotoxic Activity Assay

The cytotoxic effects of ginseng extracts, ginsenosides, and their derivatives against cancer cells, expressed as the percentage of cell viability, were determined by MTT assay described previously [17]. The assay was performed using three replicates with three independent experiments. Representative results are showed as means ± S.E.M., * *p* < 0.05, ** *p* < 0.01.

## 4. Conclusions

Based on our results, the cytotoxic potencies of the hydrolysates of RSE and total PPD-type or PPT-type ginsenosides fraction were much stronger than those of the original RSE and ginsenosides fractions. In particular the PPD-type ginsenosides hydrolysate fraction showed the most potency. Regarding the PPD-type ginsenosides, the cytotoxic potency was generally in a descending order of compound **3** > 20(*S*)-dihydroprotopanaxadiol (**2**) > PPD (**1**) > 20(*S*)-Rh2 > 20(*R*)-Rh2 ≈ 20(*R*)-Rg3 ≈ 20(*S*)-Rg3. This indicates a clear activity-structure relationship in which less polar compounds possessed higher cytotoxic activity towards the tested cancer cells. 
